# Stanniocalcin 1a regulates organismal calcium balance and survival by suppressing Trpv6 expression and inhibiting IGF signaling in zebrafish

**DOI:** 10.3389/fendo.2023.1276348

**Published:** 2023-10-26

**Authors:** Shuang Li, Helena Li, Zhengyi Wang, Cunming Duan

**Affiliations:** Department of Molecular, Cellular and Developmental Biology, University of Michigan, Ann Arbor, MI, United States

**Keywords:** Stc1a, IGF signaling, Trvp6, calcium uptake, ionocyte

## Abstract

Stanniocalcin 1 (Stc1) is well known for its role in regulating calcium uptake in fish by acting on ionocytes or NaR cells. A hallmark of NaR cells is the expression of Trpv6, a constitutively open calcium channel. Recent studies in zebrafish suggest that genetical deletion of Stc1a and Trpv6 individually both increases IGF signaling and NaR cell proliferation. While *trpv6^-/-^* fish suffered from calcium deficiency and died prematurely, *stc1a^-/-^* fish had elevated body calcium levels but also died prematurely. The relationship between Stc1a, Trpv6, and IGF signaling in regulating calcium homeostasis and organismal survival is unclear. Here we report that loss of Stc1a increases Trpv6 expression in NaR cells in an IGF signaling-dependent manner. Treatment with CdCl_2_, a Trpv6 inhibitor, reduced NaR cell number in *stc1a* ^-/-^ fish to the sibling levels. Genetic and biochemical analysis results suggest that Stc1a and Trpv6 regulate NaR cell proliferation via the same IGF pathway. Alizarin red staining detected abnormal calcium deposits in the yolk sac region and kidney stone-like structures in *stc1a* ^-/-^ fish. Double knockout or pharmacological inhibition of Trpv6 alleviated these phenotypes, suggesting that Stc1a inhibit epithelial Ca^2+^ uptake by regulating Trpv6 expression and activity. *stc1a^-/-^* mutant fish developed cardiac edema, body swelling, and died prematurely. Treatment of *stc1a^-/-^* fish with CdCl_2_ or double knockout of Trpv6 alleviated these phenotypes. These results provide evidence that Stc1a regulates calcium homeostasis and organismal survival by suppressing Trpv6 expression and inhibiting IGF signaling in ionocytes.

## Introduction

Stanniocalcins (Stcs) are evolutionarily conserved glycoproteins. The first Stc protein was discovered from the Corpuscles of Stannius (CS), an endocrine organ unique to bony fish (1, 2). Surgical removal of CS resulted in elevated blood calcium levels and the appearance of kidney stones (3–5). Injection of CS extracts or purified Stc1 reversed these effects (6). In cultured rainbow trout CSs, secretion of Stc1 was found to be regulated by external Ca^2+^ levels ([Ca^2+^]) (6). High [Ca^2+^] increased Stc1 secretion via the calcium sensing receptor (7). *In vivo*, zebrafish embryos raised in high [Ca^2+^] media showed elevated *stc1* mRNA levels (8, 9). Morpholino-based knockdown of Stc1a increased Ca^2+^ uptake and forced expression of Stc1a decreased Ca^2+^ uptake (10, 11). These and other findings have led to the notion that Stc1 is a hypocalcemic hormone in fish (2, 4, 6).

For several decades, Stc1 was considered a fish-specific hormone and even once called teleocalcin (2). Recent advances in genomics, however, have revealed that two *STC* genes are present in humans and other mammals. Human STC1 shares 61% sequence identity with fish Stc1 (12). In addition to STC1, there is a related protein (STC2), which shares ~30% identity in amino acid sequence with STC1 and contains a histidine cluster in the C-terminal region (2). Subsequent studies show that many teleost fish including zebrafish have 4 distinct *stc* genes, including *stc1a*, *stc1b*, *stc2a*, and *stc2b* (13), consistent with the notion that many teleost fish genomes underwent an additional round of genome-wide duplication (14). Published results suggest that mammalian STCs regulate somatic growth by inhibiting the insulin-like growth factor (IGF) signaling locally (15–17). IGFs act by binding to the IGF1 receptor and activating the downstream signaling cascades, including the PI3K-AKT-mTOR pathway and the RAS/RAF-MAP kinase pathway (18). In extracellular environments, IGFs are found in complexes with six types of IGF binding proteins (IGFBPs). These IGFBPs bind to IGF with an equal or greater affinity than the IGF1 receptor and therefore regulates IGF availability and biological activity (19). An important regulatory mechanism of the IGF signaling is proteolytic degradation of IGFBPs (16). Two structurally related metalloproteinases, pregnancy-associated plasma protein-a (PAPP-A) and PAPP-A2, have been shown to cleave IGFBPs and release IGFs from the IGFBP-IGF complex for IGF1 receptor binding (20). *In vivo* and biochemical studies suggest that human STC1 and STC2 function as potent inhibitors of PAPP-A and PAPP-A2 (21–23).

Recent genetic studies in zebrafish suggest that Stc1a is essential for life (24). *stc1a* ^-/-^ zebrafish developed cardiac edema around 4-5 days post fertilization (dpf). This was followed by whole body swelling and premature death (24). In zebrafish, calcium uptake is mainly carried out by Na+/H^+^-ATPase-rich (NaR) cells, one of the five types of ionocytes (25). *stc1a^-/-^* mutant larvae had significantly more NaR cells due to elevated NaR cell proliferation (24). Mechanistic analysis results show that Stc1a suppresses local IGF signaling by inhibiting Papp-aa mediated degradation of IGF binding protein 5a (Igfbp5a) in NaR cells (24, 26). A loss of Stc1a liberates IGFs from the Igfbp5a/IGF complex and increases bioavailable IGFs for IGF1 receptor binding (24, 26, 27). Addition of fish IGF-1 in excess was sufficient to increase NaR cell proliferation (26, 28). These findings suggest that the Stc1a-Papp-aa-Igfbp5a-IGF axis regulates NaR cell number and density.

A hallmark of NaR cells is the expression of Trpv6 (previously known as epithelial calcium channel or ECaC) (10, 29). Trpv6 is a constitutively open channel and it mediates continuous Ca^2+^ influx and maintains high cytoplasmic [Ca^2+^] levels (29). We have previously shown that genetic deletion of *trpv6* not only reduces calcium influx but also increases NaR cell proliferation (29). While *trpv6^-/-^* fish suffered from calcium deficiency and died prematurely, *stc1a^-/-^* fish had elevated body calcium levels but also died prematurely (24, 29). The relationship between Stc1a, Trpv6, and IGF signaling in regulating NaR cell proliferation and calcium uptake is unclear. In the current study, we provide evidence that both Stc1a and Trpv6 inhibits NaR cell proliferation by suppressing IGF signaling. Genetic deletion of Stc1a increases trpv6 mRNA levels and results in abnormal calcium deposits in the yolk sac and kidney stones. These phenotypes were rescued by inhibiting Trpv6 channel activity and by double knockout of Trpv6. Additional evidence suggests a crosstalk between Trpv6-mediated calcium signaling and IGF signaling in NaR cells and they work together to maintain calcium homeostasis and organismal survival.

## Results and discussion

**Figure 1:**
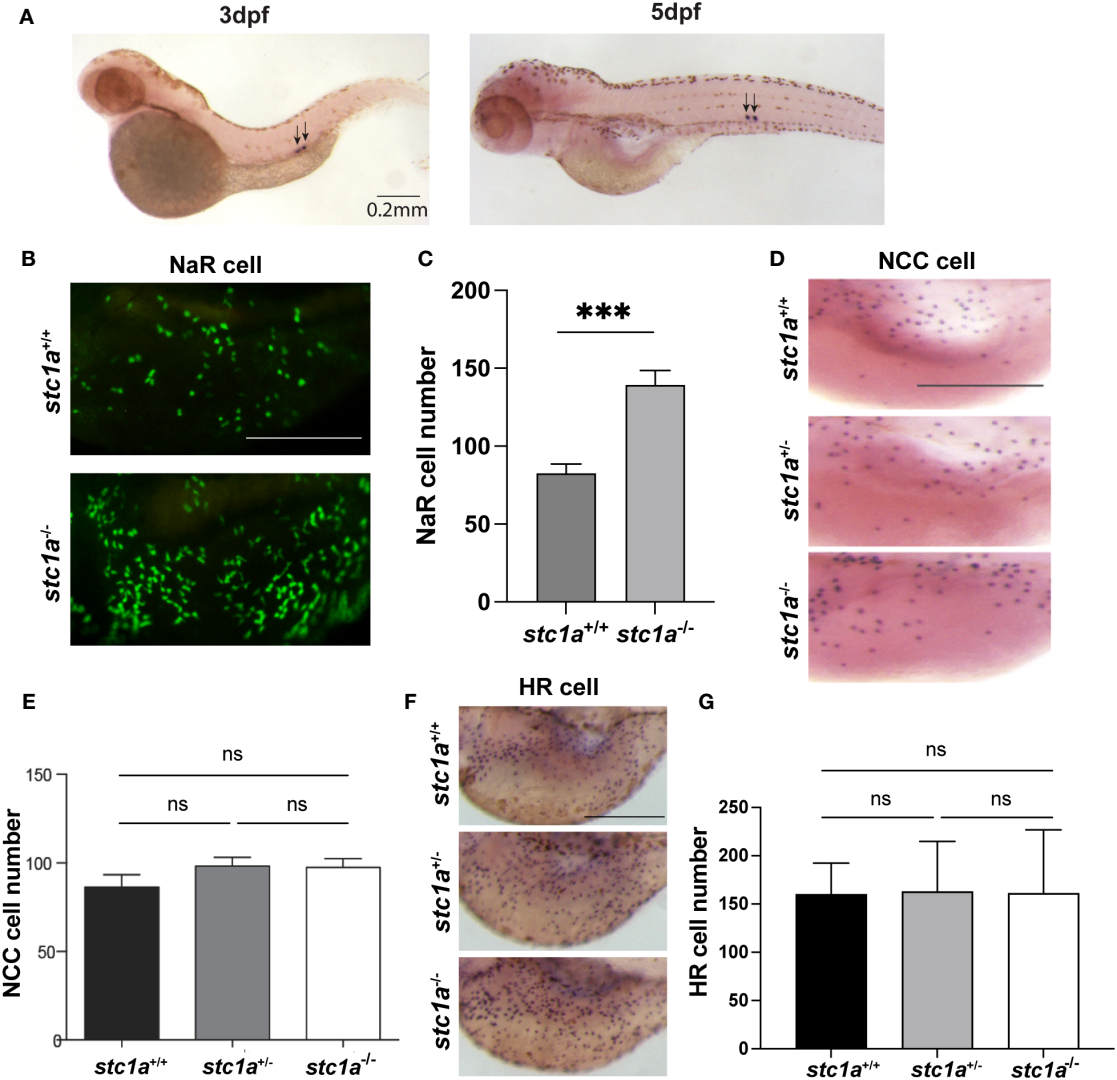
CS-derived Stc1a regulates the proliferation of NaR cells, but not other ionocyte types. **(A)** *In situ* hybridization analysis of *stc1a* mRNA expression in 3 and 5 days post fertilization (pdf) larvae. Arrows indicate the corpuscles of Stannius. **(B, C)** Loss of Stc1a increases NaR cell proliferation. *stc1a^+/+^;Tg(igfbp5a:GFP), stc1a* ^-/-^ *;Tg(igfbp5a:GFP)* embryos were raised in E3 embryo medium to 5 day post fertilization (dpf) and analyzed. Representative views are shown in **(B)**. Scale bar = 0.2 mm. The NaR cell numbers were quantified and shown in **(C)**. n = 16-19 larvae/group ***, P < 0.001. **(D, E)** NCC cells. Larvae (4 dpf) of the indicated genotypes were analyzed by *in situ* hybridization for *slc12a10.2* mRNA expression. Representative views are shown in **(D)** and quantified data in **(E)**. Scale bar = 0.2 mm. n = 4~13. ns, not statistically significant. **(F, G)** HR cells. Larvae (4 dpf) of the indicated genotypes were analyzed by *in situ* hybridization for *atpv61al* mRNA expression. Representative views are shown in **(F)** and quantified data in **(G)**. Scale bar = 0.2 mm. n = 10~15 larvae/group. ns, not statistically significant. Images shown here and in all following figures are lateral views of the yolk sac region. Anterior to the left and dorsal up. Data shown are Mean ± SEM.

Stc1a is synthesized and secreted from CS (10) (**Figure 1A**). As previously reported, genetic deletion of Stc1a resulted in a significant increase in NaR cells (24) (**Figures 1B, C**). Whether this action of Stc1a is specific to NaR cells was not clear. In this study, we determined the number of H^+^-ATPase-rich (HR) cells and Na^+^/Cl^_^ cotransporter (NCC) cells, two other ionocyte types responsible for Na^+^ uptake and Cl^-^ uptake (25). No significant difference was detected in either HR cells or NCC cells (**Figures 1D–G**) between *stc1a* ^-/-^ larvae and their siblings, suggesting the action of Stc1a is specific to NaR cells. This result is consistent with previous studies showing that Igfbp5a is specifically expressed in NaR cells, but not in other ionocyte types (30–32).

**Figure 2:**
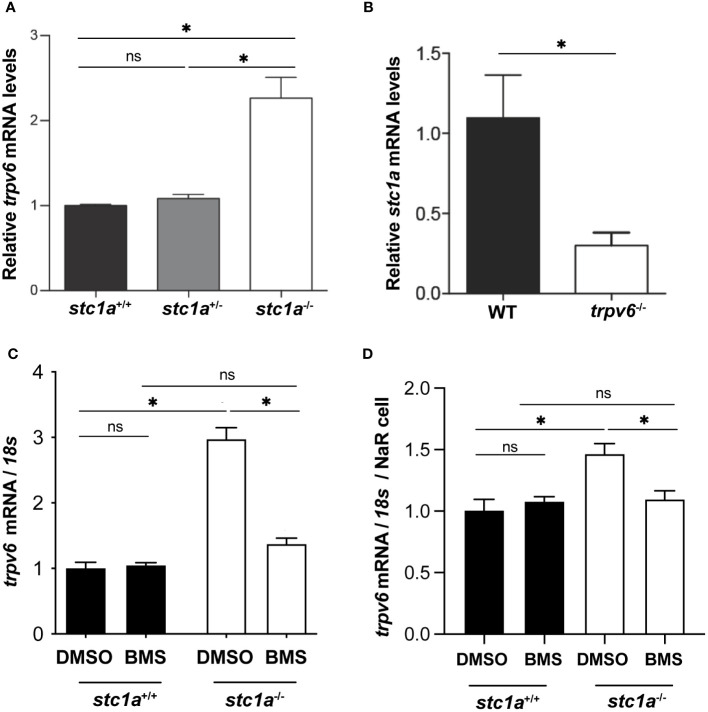
Stc1a and Trpv6 regulate each other’s expression. **(A)** Loss of Stc1a increases *trpv6* mRNA levels. Fish of the indicated genotypes were raised in E3 embryo medium. At 5 dpf, the *trpv6* mRNA levels were measured and normalized by *18S* RNA levels. n = 15~17. *, P < 0.05. **(B)** Loss of Trpv6 reduces *stc1a* mRNA levels. Embryos of the indicated genotypes were raised in E3 embryo medium. At 5 dpf, the *stc1a* mRNA levels were measured and normalized by *18S* RNA levels. n = 15~17. *, P < 0.05. **(C, D)** IGF signaling is critical in increasing Trpv6 expression in *stc1a^-/-^* fish. Larvae (4 pdf) of the indicated genotypes were treated with DMSO or BMS-754807 for one day and the *trpv6* mRNA levels were measured and normalized by *18S* rRNA **(C)**. The data were further normalized by NaR cell numbers and shown in **(D)**. Data shown are from 3 independent experiments, each containing 15 larvae/group. *, P < 0.05. ns, not statistically significant.

To test whether Trpv6 is involved in the increased NaR cell proliferation observed in *stc1a* ^-/-^ mutant fish, we measured *trpv6* mRNA levels by qRT-PCR in *Tg(igfbp5a:GFP)* fish. In *Tg(igfbp5a:GFP)* fish, NaR cells are genetically labeled by GFP expression (32), allowing quantification of NaR cells in live larvae. Compared to the siblings, *stc1a^-/-^* fish had significantly greater levels of *trpv6* mRNA (**Figure 2A**). To ascertain that the increased *trpv6* mRNA levels are not a result of increased NaR cell number in *stc1a^-/-^* fish (24), GFP-positive NaR cells were quantified and used to normalize *trpv6* mRNA levels. The *trpv6* mRNA levels/NaR cell in *stc1a* ^-/-^ were also significantly greater than the siblings (**Figure 2C**). Our finding is consistent with *in vitro* studies reporting that si/shRNA-mediated knockdown of STC1 increases TRPV6 protein levels in human CaCo2, Hela, and Caski cells (33, 34). Next, we measured *stc1a* mRNA levels in *trpv6^-/-^* fish and siblings. As shown in **Figure 2B**, *stc1a* mRNA levels were significantly lower in *trpv6^-/-^* mutant fish, suggesting that Stc1a and Trpv6 are interconnected.

Previous studies have shown that loss of Stc1a increases IGF-Akt-Tor signaling in NaR cells (24). Does the elevated IGF-Akt-Tor signaling play any role in the increase of *trpv6* mRNA expression in *stc1a^-/-^* fish? This idea was tested by treating *stc1a* ^-/-^ fish and siblings with BMS-754807, an IGF1-R inhibitor (31). As shown in **Figures 2C, D**, BMS-754807 reduced *trpv6* mRNA levels to the sibling levels, suggesting that loss of Stc1a increases Trpv6 expression via an IGF signaling-dependent mechanism. Recently, we have discovered that serum- and glucocorticoid-regulated kinase 1 (Sgk1) acts downstream in the IGF-Akt-Tor signaling pathway in NaR cells (35, 36). Studies in culture mammalian cells suggest that SGK1 up-regulates the expression of several ion channels and transporters, including the epithelial Ca^2+^ channels TRPV5 and TRPV6 (37). SGK1 influences transcription factors such as NF-κB, p53, CREB, AP-1 and FOXO3a. Future studies are needed to clarify whether Sgk1 plays a role in regulating *trpv6* expression.

**Figure 3:**
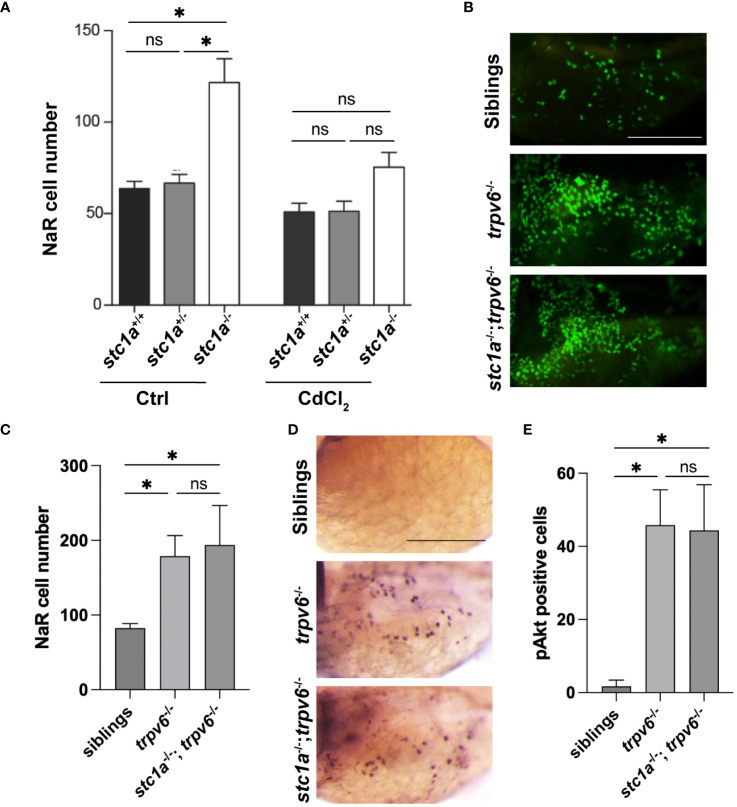
Stc1a and Trpv6 suppress NaR cell proliferation via the same IGF signaling pathway. **(A)** Inhibition of Trpv6 abolishes the elevated NaR cell proliferation in *stc1a* ^-/-^ larvae. Larvae (3 dpf) of the indicated genotypes were treated with DMSO or 10 μg/L CdCl_2_ for 2 days. GFP-labeled NaR cells were quantified and shown. n = 4~19 fish/group. *, P < 0.05. ns, not statistically significant. **(B, C)** *stc1a* ^-/-^; *trpv6* ^-/-^ double mutants phenocopy *trpv6* ^-/-^ fish. Progeny of *stc1a* ^+/-^; *trpv6* ^+/-^ in the *Tg(igfbp5a:GFP*) background were raised in E3 medium. At 5dpf, NaR cells were quantified and shown. These larvae were genotyped individually Representative images are shown in **(B)** and quantified data in **(C)**. n = 4~19 larvae/group. Scale bar = 0.2 mm. **(D, E)** Progenies of *stc1a* ^+/-^; *trpv6* ^+/-^ intercrosses were raised in E3 medium. They were subjected to whole mount immunohistochemistry using an anti-phospho-Akt antibody. Phospho-Akt positive cells in the yolk sac region were quantified. The larvae were genotyped individually afterwards. Representative images are shown in **(D)** and quantified data in **(E)**. n = 5~14 larvae/group. Scale bar = 0.2 mm.

The functional role of increased *trpv6* expression was investigated using CdCl_2_, a Trpv6 inhibitor (29). CdCl_2_ treatment reduced NaR cell number in *stc1a* ^-/-^ fish to the sibling group levels (**Figure 3A**), indicating that Stc1a suppresses NaR cell proliferation by acting through Trpv6. If this were correct, then double deletion of Stc1a and Trpv6 should phenocopy each other. Indeed, compared to the siblings, the NaR cell number of *trpv6* ^-/-^; Tg(*igfbp5a*:GFP) fish was significantly higher. Double deletion of Stc1a and Trvp6 did not cause any further increase (**Figures 3B, C**), indicating that Stc1a and Trpv6 act via the same pathway. Akt is a downstream effector of IGF signaling and has been used as a proxy of IGF signaling in NaR cells due to the lack of antibodies to detected phospho-IGF1 receptors (31). To determine whether IGF signaling is involved, we measured phosphorylated Akt levels. Few Phospho-Akt positive cells were detected in wild-type and heterozygous siblings (**Figures 3D, E**). In comparison, a robust increase in Phospho-Akt positive NaR cells was detected in *trpv6* ^-/-^ larvae (**Figures 3D, E**). The double *stc1a* ^-/-^; *trpv6* ^-/-^ mutant fish had a similar level of increase in Akt signaling as *trpv6* ^-/-^ mutant fish (**Figures 3D, E**), suggesting that Stc1a and Trpv6 inhibit NaR cell proliferation via the same IGF signaling. It is worthy to point out the difference in the two approaches used to inhibit Trpv6 function/activity in this study. In **Figure 3A**, CdCl2 treatment was carried out in *stc1a^-/-^* fish. These fish have a functional Trpv6 and at elevated levels. In this setting, CdCl2 treatment inhibited Trpv6-mediated calcium influx and resulted in reduced NaR cell proliferation, supporting the conclusion that Stc1a acts via Trpv6 to suppress NaR cell proliferation. In comparison, the experiment shown in **Figures 3B, C** used *trpv6^-/-^* mutant larvae. In this genetic deletion model, there is no functional Trpv6 (29). Loss of Stc1a did not cause a further increase in NaR cell proliferation in the absence of a functional Trpv6. This result is in agreement with our conclusion.

**Figure 4:**
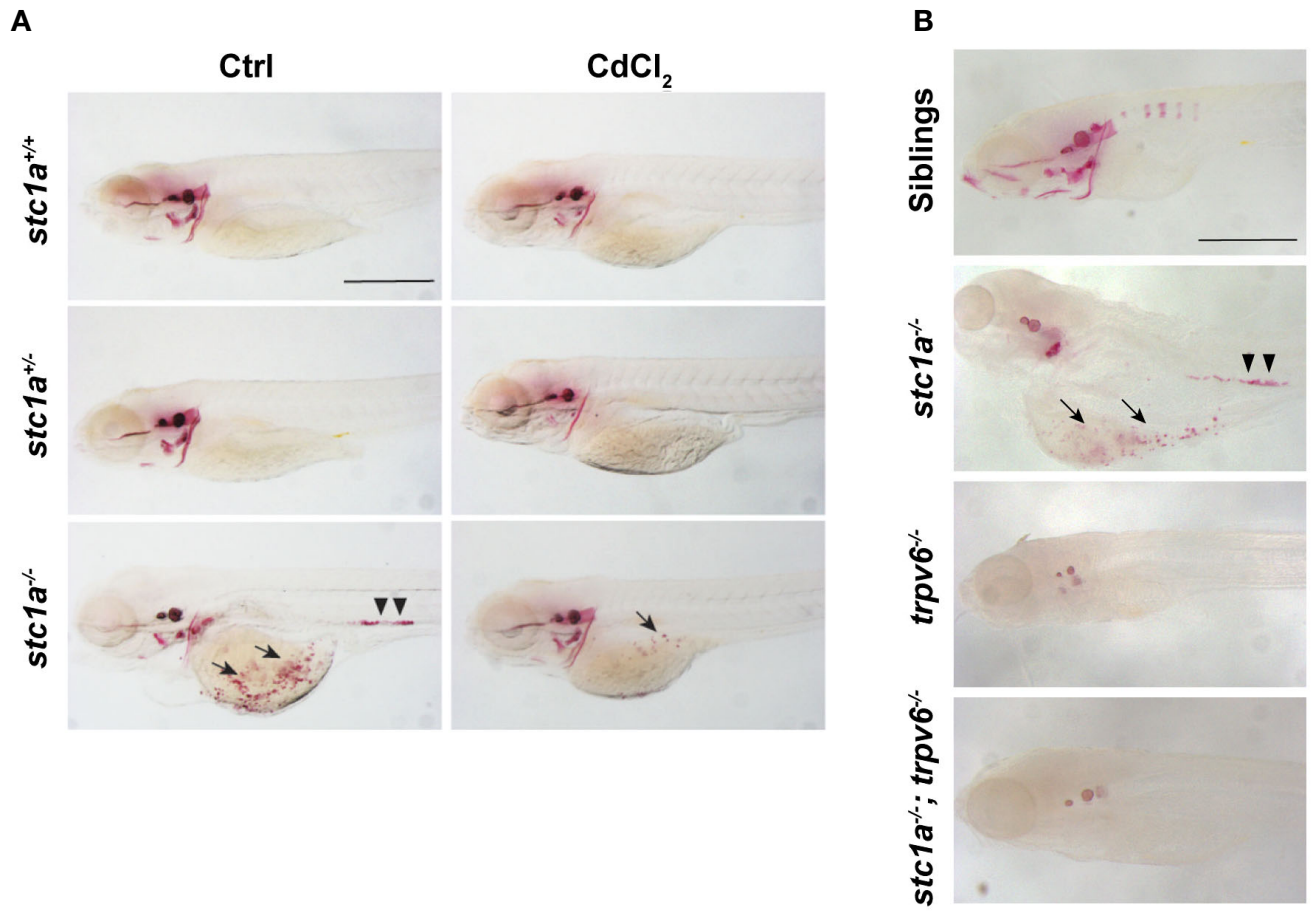
Loss of Stc1a results in abnormal calcium deposits in a Trpv6-depndent manner. **(A)** Larvae (3 dpf) of the indicated genotypes treated with or without 10 μg/L CdCl_2_ for 2 days. They were subjected to Alizarin red staining at 5 dpf. Representative images are shown. Note the ectopic calcified structures in the yolk sac region (arrow) and kidney stones (arrow heads) in the mutant fish. Scale bar = 0.5 mm. **(B)** Alizarin red staining analysis of 7 dpf zebrafish larvae of the indicated genotypes. Representative images are shown. Note the ectopic calcified structures in the yolk sac region (arrow) and kidney stones (arrow heads) in the mutant fish. Scale bar = 0.2 mm.

It has been documented half a century ago that removal of CS resulted in increased body calcium contents and the appearance of kidney stones (4). This has been attributed to the loss of Stc1. This notion, however, has not been tested genetically due to the lack of a stable genetic mutant. We visited this issue using the *stc1a* ^-/-^ mutant fish. Compared to their wild-type and heterozygous siblings, abnormal calcium deposits were observed in the yolk sac region where NaR cells are located (**Figure 4A**). Highly calcified stone-like structures were also observed in the renal tube (**Figure 4A**). In a previous report, we have quantified the calcium levels in *stc1a^-/-^* mutants and sibling embryos and found that *stc1a^-/-^* fish had significantly elevated calcium levels (24). Taken together, these data suggest that a permanent loss of Stc1a results in calcium imbalance and the development of kidney stones, essentially recapitulating the classical experiment results reported by Pang in the 1970s (4) using molecular genetics in zebrafish. Are these abnormal calcium deposits and kidney stones observed in *stc1a* ^-/-^ larvae related to the increased *trpv6* gene expression (**Figure 2**)? To address this question, we treated the fish with CdCl_2_. CdCl_2_ markedly reduced the calcified structures in the yolk sac region and in the renal tubes (**Figure 4A**). This was investigated further using double mutant fish. Alizarin red staining showed that the abnormal calcified structures were not observed in the *stc1a* ^-/-^; *trpv6* ^-/-^ double mutant fish. *trpv6* ^-/-^ fish had markedly reduced staining as well (**Figure 4B**). These results suggest that Stc1a inhibits epithelial Ca^2+^ uptake by regulating Trpv6 expression and activity.

**Figure 5:**
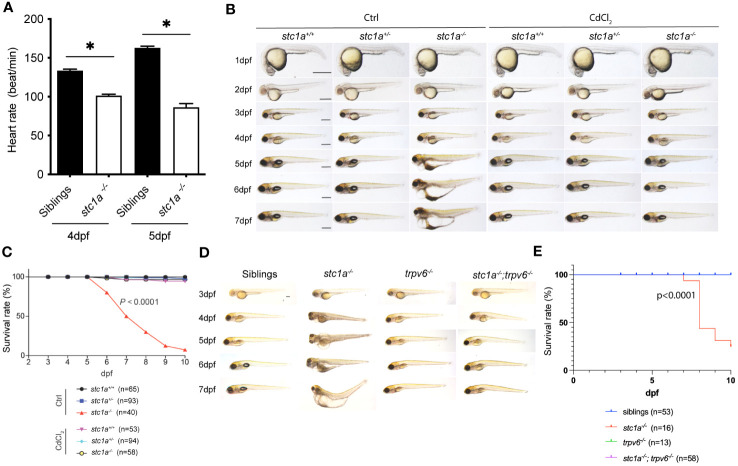
Pharmacological inhibition and double deletion of Trpv6 rescues cardiac edema and body swelling, and delays premature death of *stc1a* ^-/-^ fish. **(A)** Loss of Stc1a reduces heartbeat rate. Heartbeat rate *stc1a^-/-^* and siblings was determined and shown. *, P < 0.05. n = 9~17. **(B)** Gross morphology of fish of the indicated genotypes at the indicated time. Progeny of *stc1a^+/-^* intercrosses were raised in E3 embryo medium and treated with or without 10 μg/L CdCl_2_ from 3 dpf until the indicated time. Fish were genotyped individually. Representative views of the indicated genotypes at the indicated stages are shown and survival curve shown in **(C)**. Scale bar = 0.5 mm. P < 0.0001 by log-rank test. **(D, E)** Gross morphology of fish of the indicated genotypes at the indicated time. Representative views at the indicated stages are shown and survival curve shown in **(E)**. Scale bar = 0.2 mm. P < 0.0001 by log-rank test.

At 4-5 dpf, *stc1a* ^-/-^ mutants developed cardiac edema and this was followed by whole body swelling and premature death (24). In this study, we detected a significant reduction in heart rates (**Figure 5A**). These phenotypes are very different from the mouse model. Stc1^-/-^ null mice grew normally with no notable anatomical abnormalities (38). These differences among species may relate to their distinct physiology and different habitats. Zebrafish Stc1a is expressed and secreted from CS glands in a calcium concentration-regulated manner (10, 24, 39). Mice, however, do not have CS glands and *Stc1* gene is expressed in many tissues and likely acts locally as a PAPP-A/PAPP-A2 inhibitor (2, 17). Mouse Stc1 does not appear to affect calcium homeostasis because Stc1 knockout mice had normal circulating calcium levels and normal Vitamin D3 response (38). Mice and other territorial animals take up Ca^2+^ from food and drinks. Zebrafish live in freshwater, a hypoosmotic aquatic environment (40). Zebrafish actively regulate their body osmolarity by maintaining ion water balance. They use ionocytes to uptake salts. At the same time, zebrafish remove the excess osmotic water by producing and excreting large volumes of diluted urine and reabsorbing ions in the kidney (39, 40). Although zebrafish nephrons begin to form, efficient glomerular filtration and ion re-absorption begin around 4-5 dpf (39, 41, 42). The cardiac edema and body swelling phenotypes observed in *stc1a* ^-/-^ mutant fish begin to manifest around 4-5 dpf. These led us to speculate that elevated epithelial Ca^2+^ uptake and impaired renal function may result in the accumulation of osmotic water, which lead to the progressive development of edema and swelling. If this were correct, then pharmacological or genetic blockade of Trpv6-mediated Ca^2+^ uptake should rescue the *stc1a* mutant fish. Indeed, treatment of *stc1a^-/-^* fish with CdCl_2_ alleviated the edema and body swelling phenotype (**Figure 5B**). While *stc1a^-/-^* fish died between 6 to 10 dpf, there was no death in the CdCl_2_ treated group until 10 dpf (**Figure 5C**). The role of Trvp6-mediated epithelial Ca^2+^ uptake was tested further by double knocking out *stc1a* and *trpv6*. As shown in **Figures 5D, E**, no cardiac edema or body swelling was observed in *stc1a* ^-/-^; *trpv6* ^-/-^ double mutant larvae. All *stc1a* mutant larvae lacked inflated swimming bladders (**Figure 5D**). This phenotype was rescued by CdCl_2_ treatment (**Figure 5A**) but not by double deletion of *stc1a* ^-/-^ and *trpv6* ^-/-^ (**Figure 5C**). The reason is not clear at this time. We have reported that the premature death can be rescued by reducing NaR cell number via pharmacological inhibition of the IGF1 receptor and Tor or by double deletion of *igfbp5a* or *papp-aa* in the *stc1a*-/- background (24). Since Stc1a and Trpv6 inhibit NaR cell number via the same IGF signaling, we tested the possible role of Trpv6 in zebrafish survival. While many *stc1a* ^-/-^ fish died between 7 to 10 dpf, no death was detected in *stc1a* ^-/-^; *trpv6* ^-/-^ fish, *trpv6* ^-/-^ or siblings until 10 dpf (**Figures 5D, E**). These data suggest that the increased calcium uptake due to the combinatory effects of more NaR cells and great Trpv6 expression/NaR cell may cause ion water imbalance and premature death of *stc1a* ^-/-^ fish.

**Figure 6:**
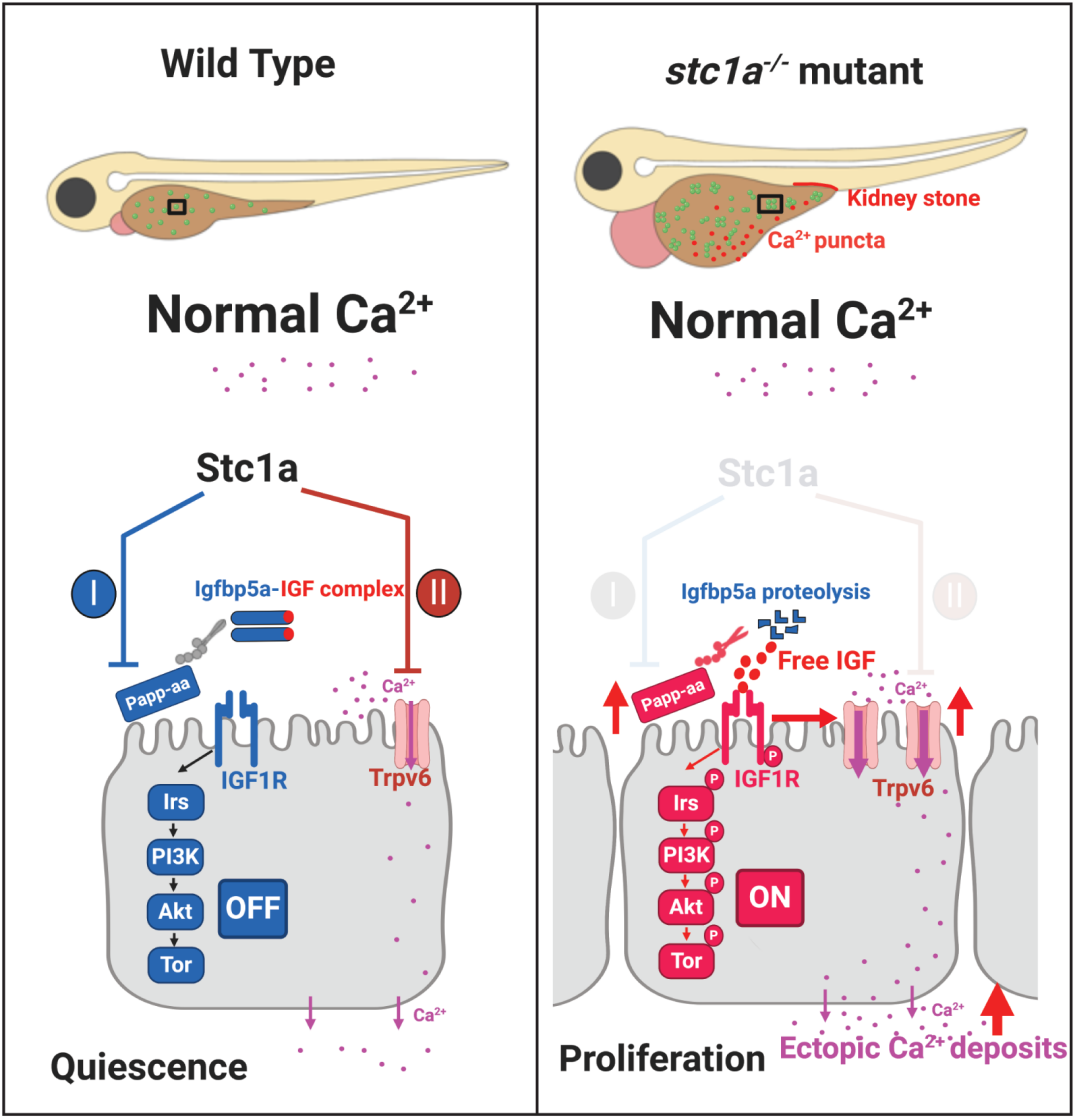
A proposed model. Stc1a plays dual roles in ionocytes. Stc1a suppresses local IGF signaling and inhibits NaR cell proliferation by inhibiting Papp-aa-mediated Igfbp5a degradation. Stc1a also inhibits Trpv6 expression and activities. These two functions are linked. Trpv6-mediated calcium signaling inhibits IGF signaling, while IGF signaling upregulates Trpv6 expression. A loss of Stc1a reactivates IGF-PI3 kinase-Akt-Tor signaling in NaR cells and increased NaR cell proliferation. In addition, Trpv6 expression and Trpv6-mediated calcium uptake in each NaR cell are elevated in the *stc1a^-/-^* mutant fish. These changes contribute to abnormal calcium deposits in the yolk sac region and kidney and to the developemnt of cardiac edema, body swelling, and premature death phenotypes.

In summary, the results of this study have provided genetic and biochemical evidence that Stc1a regulates calcium homeostasis and organismal survival by playing dual roles in ionocytes (**Figure 6**). Stc1a suppresses NaR cell proliferation via its reported role in inhibiting Papp-aa-mediated local Igfbp5a degradation (24, 26). Stc1a also inhibits Trpv6 expression and/or Trpv6-mediated calcium uptake (**Figure 6**). These two functions are linked. While Trpv6-mediated calcium uptake inhibits IGF signaling, IGF signaling upregulates Trpv6 expression and stimulates NaR cell proliferation (**Figure 6**). A loss of Stc1a results in a reactivation of IGF-PI3 kinase-Akt-Tor signaling in NaR cells, which stimulates NaR cell proliferation and increase NaR cell number and calcium uptake. In addition, loss of Stc1a also increases Trpv6 expression and Trpv6-mediated calcium uptake. These changes contribute to abnormal calcium deposits in the yolk sac region and in the kidney, the development of edema, body swelling, and premature death (**Figure 6**). The current study also reveals a feedback loop from Trpv6 to Stc1a. While loss of Stc1a increases Trpv6 expression in NaR cells, loss of Trpv6 expression decreases Stc1a expression in CS. These findings provide new insights into our understanding of Stc1/STC1. At present, the biochemical pathways that lead to the formation of ectopic calcium deposits in the yolk sac region and in renal tubes found in the *stc1a* ^-/-^ mutant fish are not clear. In the adult stages, NaR cells are distributed mainly in the gills and kidney. Because *stc1a^-/-^* mutant fish die prematurely, the function of Stc1a in adult physiology is not clear. A conditional knockout fish model will be needed to elucidate Stc1a’s actions in the adult gills, kidney, and intestine. In addition to *stc1a*, zebrafish have 3 other *stc* genes. Future studies will be needed to elucidate their functions and the relationship among these genes.

## Materials and methods

### Zebrafish

The experiments were conducted in accordance with the guidelines approved by the Institutional Committee on the Use and Care of Animals, University of Michigan. Zebrafish were raised, maintained, crossed, and staged in accordance with the standard zebrafish husbandry guidelines (43). Embryos and larvae were raised at ~28°C in the standard E3 embryo medium. To inhibit pigmentation, 0.003% (w/v) N-phenylthiourea was added to these medium. The *Tg(igfbp5a:GFP)* fish line, *Tg(ifbp5a:*GFP);*stc1a^+/-^*, and *Tg(igfbp5a:*GFP);*trpv6^+/-^* fish line were generated in previous studies (24, 26, 27, 29). Double mutant fish were generated by crossing these lines.

### Genotyping

Fish larvae or adult fish fin were digested in 100 μl SZL buffer (50 mM KCl, 2.5 mM MgCl2, 10 mM tris-HCl (pH 8.3), 0.45% NP-40, 0.45% Tween 20, 0.01% gelatine) and proteinase K (100 μg/ml) at 60°C for 2 hours. The reaction was stopped by 15-minute heat treatment (95°C). The genotyping was performed by PCR using the digestion mixture as a template as previously reported (24, 29).

### Morphological analysis and heart rate

Heat rate was determined by counting heartbeat manually under a stereomicroscopy. For morphology imaging, embryos and larvae were briefly anesthetized with Tricaine and mounted in 1.5% agarose and imaged. Bright field images were acquired using a stereomicroscope (Leica MZ16F, Leica, Wetzlar, Germany) equipped with a QImaging QICAM camera (QImaging, Surrey, BC, Canada). After imaging, embryos and larvae were washed and returned to the E3 embryo medium.

### Immunostaining, *in situ* hybridization, and Alizarin red staining

Immunostaining of phospho-Akt was performed as previously described (31, 44). Briefly, zebrafish larvae were fixed overnight in 4% paraformaldehyde. They were dehydrated in methanol for two hours at -20°C and washed with PBST (Triton 0.1%). After incubated with in PBST containing 5% horse serum for 1.5 hours at 4°C. The larvae were rinsed and incubated overnight with an antibody against phospho-Akt at 4°C. They were washed with PBST and 5% HS in PBST. The larvae were incubated with an anti-rabbit HRP antibody (Jackson ImmunoResearch, West Grove, PA, USA) for 3 hours at room temperature and visualized by nickel-diaminobenzidine staining. Whole mount *in situ* hybridization was performed as previously reported (30, 31, 45). Calcified tissues were detected by Alizarin red staining as reported previously (27).

### qRT-PCR

Total RNA was extracted from pooled zebrafish embryos and larvae as reported (46). RNA was reverse transcribed to cDNA using oligo(dT)18 primer and M-MLV (Promega). qPCR was performed using SYBR Green (Bio-Rad) on a StepONEPLUS real-time thermocycler (Applied Biosystems). The expression level of a target gene transcript was normalized by 18S RNA level. The following primers were used: trpv6-qPCR-F: 5’- GGACCCTACGTCATTGTGATAC-3’, trpv6-qPCR-R: 5’-GGTACTGCGGAAGTGCTAAG-3’, 18s-qPCR-F: 5’-AATCGCATTTGCCATCACCG-3’, and 18s-qPCR-R: 5’-TCACCACCCTCTCAACCTCA-3’.

### Drug treatment

All drugs were dissolved in DMSO and further diluted in double deionized water as previously reported (24, 31). Drug solutions were changed daily.

### Statistical analysis

Statistical tests were determined using GraphPad Prism 8 software (GraphPad Software, Inc.,San Diego, CA). Values are shown as means ± SEM. Unpaired two-tailed t-test, Chi-square test, log-rank test and one-way ANOVA followed by Tukey’s multiple comparison test were used to determine statistical significance of experimental groups. A p-value less than 0.05 was accepted as statistically significant.

## Data availability statement

The original contributions presented in the study are included in the article/supplementary material. Further inquiries can be directed to the corresponding author.

## Ethics statement

The animal study was approved by Institutional Committee on the Use and Care of Animals, University of Michigan. The study was conducted in accordance with the local legislation and institutional requirements.

## Author contributions

SL: Writing – review & editing, Data curation, Formal Analysis, Investigation, Visualization. HL: Data curation, Formal Analysis, Investigation, Visualization, Writing – review & editing. ZW: Data curation, Formal Analysis, Investigation, Visualization, Writing – review & editing, Validation. CD: Writing – review & editing, Conceptualization, Funding acquisition, Project administration, Resources, Supervision, Writing – original draft.
